# A Novel Single-Loop Mechanism for Neck Rehabilitation

**DOI:** 10.3390/biomimetics10120814

**Published:** 2025-12-04

**Authors:** Raffaele Di Gregorio

**Affiliations:** Laboratory of Mechatronics and Virtual Prototyping (LaMaViP), Department of Engineering, University of Ferrara, Via Saragat 1, 44122 Ferrara, Italy; raffaele.digregorio@unife.it; Tel.: +39-053-297-4828

**Keywords:** neck rehabilitation, neck brace, spherical mechanism, kinematic analysis, dimensional synthesis

## Abstract

Trauma, amyotrophic lateral sclerosis (ALS), and head and neck cancer (HNC), which cause neck pain, are only some of the possible issues requiring suitable therapy for alleviating or even healing the neck dysfunctions they cause. Static and dynamic neck braces are commonly employed in therapies for neck recovery and in the necessary measurements to quantify neck impairment or to set up a suitable therapy. Serial and parallel mechanisms, among others, have been proposed for neck braces. Here, a novel single-loop spherical mechanism is proposed for a possible neck brace. Its kinematics and mobility analyses are presented with reference to their specific applications in a neck brace. Then, dimensional synthesis with a set of neck brace’s kinematic requirements is addressed to compute the geometric constants that guarantee an orientation workspace similar to that of the human neck. The presented analyses and syntheses show that the new proposal is effective and can alleviate some concerns about already-proposed mechanisms for neck braces.

## 1. Introduction

Neck dysfunctions result from musculoskeletal disorders and/or neurologic issues caused by diseases (e.g., amyotrophic lateral sclerosis (ALS) [1] and head and neck cancer (HNC) [2]) or accidents (e.g., whiplash due to an impact [3]). Most of these causes yield neck pain [4] and a reduction in the Range Of Motion (ROM) of the cervical spine (i.e., of the relative motion between the head and the shoulders), since they ultimately lead the patient to reduce their neck’s ROM to avoid pain. Neck pain globally affected 203 million people [5] in 2020 (i.e., 2450 people per 100,000 population) and has a high social cost [6] due to absences from work and health care requirements. These data show that neck pain is one of the most common musculoskeletal disorders.

The application of mechanical actions to the patient’s neck by means of neck braces [3] is one of the physical therapies that is often necessary to either alleviate or even heal neck dysfunctions. Indeed [4,7], immobilization, stretching, or passive mobilization of the neck yield benefits in many cases. Neck braces are also used for the measurements [8,9] that are necessary to quantify neck impairment or to set up suitable therapy.

Static and dynamic neck braces have been proposed for neck therapies. Static neck braces are mainly collars that allow for the immobilization of the neck at a fixed posture [3,10] to match the needs of cervical spine injury (CSI) healing and of post-cervical spine surgery [11]. Conversely, dynamic neck braces are mechanisms that allow for a guided relative motion between the head and the shoulders and, in general, can be implemented in any type of neck physical therapy.

Serial [9,12,13] and parallel mechanisms [8,14,15,16,17,18,19,20] have been proposed such as neck braces both for Wearable Assistive Devices (WADs) and for outpatient therapies. Such mechanisms connect a frame, fixed either to the patient’s shoulders (e.g., through a vest) or to the seat the patient is sitting on, to a distal link (end-effector) fixed to the patient’s head (e.g., through a harness surrounding the forehead (i.e., frontal, parietal, and occipital bones) or a chin carrier suitably connected to the back of the head). In serial (parallel) mechanisms, one kinematic chain (a number of kinematic chains (limbs)) with links connected in series joins (simultaneously) these two ending links. In general, Serial Mechanisms (SMs) allow for a ROM that is wider than that of Parallel Mechanisms (PMs), but they are less precise and stiff than PMs. In terms of kinetostatic performance, a good compromise might be a PM with a reduced number of limbs.

The natural motion that the neck allows for the head with respect to the shoulders is a spatial motion with six Degrees Of Freedom (DOFs) where the three translational DOFs have limited ROMs and the three rotational DOFs have large ROMs [21,22]. Consequently, a three-DOF spherical or quasi-spherical motion [23] can approximately mimic this motion. Accordingly, dynamic neck braces with less than three DOFs [13,19] perform only specific tasks, whereas those with three [8,12,16,17,20] or more (up to six) [9,14,15] are general-purpose, and they are conceived to implement any task.

A reduced number of DOFs facilitates the design of PMs with a reduced number of limbs, since having only one actuated pair per limb, which is located on the frame, is a common design choice. Consequently, in the literature, neck braces based on PM types with three DOFs (3RRS [8], 3RPS [16,17], and 3RXS [20] 1) are much more common than those with six DOFs (6SPS with a 6-3 [14] and 6-6 [15] limb arrangement). The limb number can be further reduced to two by adopting three-DOF single-loop architectures with two actuated joints on the frame and a third near the frame. Here, the use of these types of architectures as neck braces is investigated and a novel type of dynamic neck brace is proposed together with the kinematic analysis and dimensional synthesis thereof. The proposed neck brace can be sized to generate both spherical and quasi-spherical motion since it is based on a non-overconstrained single-loop three-DOF spatial architecture.

This paper is organized as follows. Section 2 provides the necessary background materials, identifies the design requirements of a general-purpose dynamic neck brace, and presents the architecture of the novel dynamic neck brace together with its kinematic and singularity analyses. Then, Section 3 uses the relationships deduced in the previous section to address the dimensional synthesis of the proposed orthosis and the workspace analysis of the mechanism. Finally, Section 4 discusses the obtained results, and Section 5 concludes the paper.

## 2. Materials and Methods

Analysis of the neck’s natural motion is central to the definition of the design requirements of a dynamic neck brace. In this section, neck biomechanics is firstly briefly recalled to identify these requirements. Then, the selection of the proposed single-loop architecture is discussed with reference to the literature on dynamic neck braces and on spherical 3-DOF non-overconstrained PMs. Eventually, the kinematics of the proposed mechanism is studied to deduce all the relationships needed to size it according to given motion requirements.

### 2.1. Summary of Neck Biomechanics and Identification of Neck Brace Requirements

The kinematic constraint that the neck imposes on the relative motion between the head and the shoulders in healthy subjects and patients with neck injuries has been analyzed in many papers (see, for instance, [24,25,26,27]) and textbooks (see, for instance, [21,22]).

**Figure 1:**
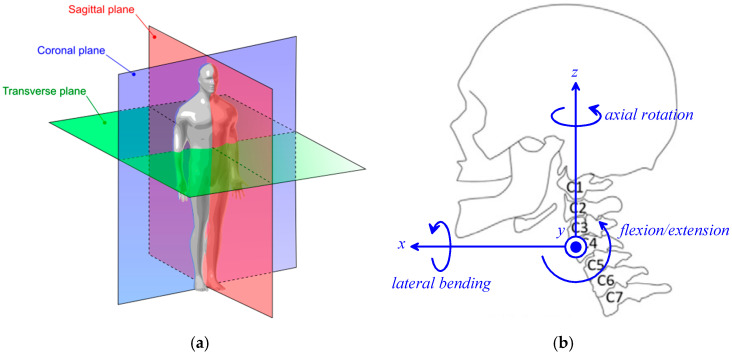
Neck movements: (**a**) cardinal planes of the human body (reproduced from chapter 1.4D of Ref. [28]), (**b**) sagittal-plane view of skull and cervical spine in the standard anatomical (neutral) position [28] (the *xz*-coordinate plane coincides with the sagittal plane; the *xy*-coordinate plane and the *yz*-coordinate plane are parallel to the transverse plane and the coronal plane, respectively; adapted from [9]).

The head displacements are measured by taking as pose reference the *standard anatomical position* (see chapter 1.4A of Ref. [28]), also named *Frankfurt plane*, and hereafter referred to as *neutral position*. The neutral position of the head (Figure 1b) is the one assumed by the skull when the subject is standing upright and looking straight ahead (Figure 1a) with the orbitales (eye sockets), lower margins of the orbits, and the poria (ear canal upper margins) all lying on the same horizontal plane, which is parallel to the transverse plane (see Figure 1a). In this pose, introducing the two coincident Cartesian reference systems shown in Figure 1b, one fixed to the skull and the other fixed to the shoulders, makes it possible to define the following head displacements (note that the xz-coordinate plane coincides with the sagittal plane (see Figure 1a) and the coordinate planes xy and yz are parallel to the transverse and the coronal plane, respectively):

- Counterclockwise rotation around the *x*-axis, named Right(+)/Left(−) Lateral Bending;
- Translation along the *x*-axis, named Protraction(+)/Retraction(−);
- Counterclockwise rotation around the *y*-axis, named Flexion(+)/Extension(−);
- Translation along the *y*-axis, named Left(+)/Right(−) translation;
- Counterclockwise rotation around the *z*-axis, named Left(+)/Right(−) Axial Rotation;
- Translation along the *z*-axis, named Upward(+)/Downward(−) translation.

From a mechanical point of view, the cervical spine consists of seven links (see Figure 1b), the vertebrae C1 (atlas), C2 (axis), C3, …, C7, that connect the occipital bone (O) of the skull to the first vertebra of the thoracic spine, T1. The intervertebral joints O-C1 and C1–C2 have a structure that is different from the remaining joints. Nevertheless, in general, all the intervertebral joints can be considered as spherical pairs with a limited mobility that, in the O-C1, is very limited in all the coordinate planes and, in the C1-C2, is much larger in the horizontal plane (axial rotation). Consequently, the number of DOFs of the cervical spine is higher than six. Even though the cervical spine is a redundant spatial kinematic chain, the limited ROM of each intervertebral joint makes the translation of the skull with respect to the shoulders an unnatural motion, limited to few centimeters along the x axis [29] and negligible along the y and z axis, which, in practice, reduces the actual number of DOFs mainly to three rotations. Mechanisms that try to replicate the kinematic structure of the cervical spine have been presented in the literature (see, for instance, [30,31]).

The fact that rotations are prevalent in neck motion has led to the characterization of head mobility by evaluating only these three rotations using different measurement techniques [32]. Almost all the reported measurements consider these three movements as independent planar motions occurring in the three coordinate planes (i.e., the sagittal, horizontal, and coronal planes for flexion/extension, axial rotation, and lateral bending, respectively), and give their extreme values when performed starting from the neutral position. Table 1 summarizes these values as presented in [21] and Table 2 provides the contribution to the ROM due to each intervertebral joint as presented in [22]. These values suggest the adoption of the values reported in Table 3 for the ROM of a dynamic neck brace that has to match the motion needs of nearly all potential patients. Also, if a 3-DOF spherical/quasi-spherical mechanism is adopted, the fact that the neck anatomy allows very limited translations leads the designer to impose that the spherical motion center (SMC) of the brace be located on the cervical spine more or less in the middle (i.e., at the level of vertebra C4 (see Figure 1b). Indeed, this choice minimizes the relative translation between two adjacent vertebrae that occurs when the patient’s neck has to follow the motion imposed by the dynamic neck brace. The best position of the SMC could also be determined through self-alignment techniques similar to the ones presented in [33,34], when the patient starts wearing the brace, by making the patients move their head with a brace that follows these movements with the calibration joints unlocked. Below, Section 4 discusses how to locate the SMC in greater depth.

### 2.2. The RRU-RRS Mechanism

From an architectural point of view, as explained above, a 3-DOF, spherical, non-overconstrained, single-loop mechanism is a good compromise, combining structural simplicity with good stiffness and a sufficiently large workspace, as well as the potential for quasi-spherical motion. The type synthesis of spherical 3-DOF mechanisms has been addressed in the literature [35,36,37,38,39], and single-loop architectures have been identified.

The Chebychev–Grübler–Kutzbach criterion and Euler’s formula [40,41] applied to single-loop mechanisms yield

(1) $$\left.\begin{array}{l} l=f(n-1)-\sum_{i=1,f-1}(f-i)c_{i}\\ 1=\sum_{i=1,i\max}c_{i}-n+1\Rightarrow n=\sum_{i=1,f-1}c_{i}+\sum_{i=f,i\max}c_{i} \end{array}\right\}\Rightarrow l=\sum_{i=1,f-1}i\;c_{i}+f\left(\sum_{i=f,i\max}c_{i}-1\right)$$

where *l* is the mechanism’s DOF number, *n* is the link number, *c_i_* is the number of joints with *i* DOFs, and *f* is the DOF number of the free rigid body, which is equal to 6 for spatial motion and to 3 for spherical motion. A single-loop spherical mechanism, that is, with *l* ≥ 1 for *f* = 3 in Equation (1), is overconstrained if Equation (1) gives *l* ≤ 0 for *f* = 6, and non-overconstrained otherwise. This means that, if the geometric conditions that impose the spherical motion of all the links are not satisfied, overconstrained mechanisms become isostatic (*l* = 0) or hyperstatic (*l* < 0) structures and jam, whereas non-overconstrained ones remains hypostatic (*l* ≥ 1) structures and keep moving with a spatial quasi-spherical motion. Consequently, a single-loop 3-DOF neck brace requires a spherical non-overconstrained architecture yielding *l* = 3 for *f* = 6 in Equation (1) to enable the distal link to execute both spherical and quasi-spherical motion. Moreover, in such a mechanism, each of the two limbs simultaneously connecting the frame and the distal link must have a *connectivity* of at least 3. By extension from [42], the *limb connectivity* is defined as the number of DOFs that the distal link would possess, with respect to the frame, if it were connected to the frame only by that limb. Indeed, the number of DOFs of the relative motion between the distal link and the frame cannot be higher than the minimum value of the connectivity of the limbs.

**Figure 2:**
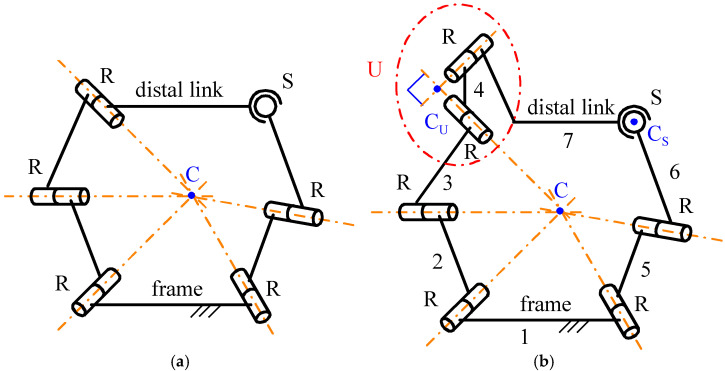
Kinematic schemes of the spherical mechanisms of the RRR-RRS (**a**) and RRU-RRS (**b**) types, where point C is the SMC.

Based on the considerations outlined above, the 3-DOF spherical, non-overconstrained, single-loop architecture proposed here was derived from a spherical architecture of type n RRR-RRS (Figure 2a) where all of the R-pair axes share a common intersection point (point C in Figure 2a). Having a common intersection point shared by all of the R-pair axes is the geometric condition that imposes the spherical motion on all of the links, with this point serving as the SMC. This particular RRR-RRS architecture consists of two limbs, one of type RRR and the other of type RRS, that simultaneously connect the distal link to the frame. The RRR limb, which has a connectivity of three, makes the distal link perform only spherical motion with respect to the frame, since the axes of its three R pairs share a common point. Conversely, the RRS limb, which has a connectivity of five, only limits/controls the spherical motion imposed by the RRR limb. For this architecture, Equation (1) gives five DOFs for *f* = 3, but only three DOFs are possible for the distal link due to the RRR limb (i.e., the remaining two DOFs are idle and could be eliminated by replacing the S pair with another R pair whose axis passes through the SMC), but, unfortunately, only two DOFs for *f* = 6. Therefore, if the geometric condition that guarantees the spherical motion is violated, this mechanism will perform a quasi-spherical motion with only two DOFs, which are not sufficient for its application in a neck brace. This problem is solvable by adding an idle R pair at the end, adjacent to the distal link, of the RRR limb whose axis is perpendicular to the axis of the third R pair of this limb (Figure 2b). The resulting architecture is of type RRU-RRS (Figure 2b), where U stands for universal joint. The U joint of the RRU limb is constituted by two mutually orthogonal R pairs: the first one is not adjacent to the distal link, with axis passing through the SMC of the old RRR limb, and the second one is adjacent to the distal link. For this new mechanism, the following statement gives the geometric condition that guarantees the spherical motion of all links.

**Statement.**  *In an RRU-RRS mechanism, if, out of singularities, the axes of the first three R pairs of the RRU limb and of the two R pairs of the RRS limb share a common intersection point, all the links are constrained to perform a spherical motion with the common intersection point being the SMC*.

The proof of this statement is as follows.

**Proof.**  In the RRU limb, the two links that join the three joints of this limb and the cross link of the U joint (link 4 in Figure 2b), when manufactured and assembled so that the axes of the first three R pairs share a common intersection point, physically keep this point shared during motion. Therefore, they are physically constrained to perform a spherical motion with this shared point as the SMC, hereafter termed SMC_RRU_, and the center of the U joint (point C_U_ in Figure 2b), which is the intersection point of the axes of the two mutually orthogonal R pairs constituting the U joint, must always move on a sphere centered at SMC_RRU_.

Moreover, in the RRS limb, if the link that joins the two R pairs is manufactured such that the axes of these two R pairs share a common intersection point, it physically keeps this point shared during motion. Therefore, the two links that join the three joints of this other limb are physically constrained to perform a spherical motion with this intersection point as the SMC, hereafter termed SMC_RRS_, which constrains the center of the S joint (point C_S_ in Figure 2b) to move on a sphere centered at SMC_RRS_.

If such RRU and RRS limbs are assembled in an RRU-RRS mechanism such that SMC_RRU_ coincides with SMC_RRS_, five of the six mobile links of the mechanism must perform a spherical motion with the same SMC and the two points C_U_ and C_S_ must move on concentric spheres that have this SMC as their center. Therefore, under this condition, if also the distal link, which is the sixth mobile link, performs a spherical motion with the same SMC, the proof of the above statement is complete.

Such a demonstration can be given as follows. The two points C_U_ and C_S_ are points at rest with respect to the distal link and can be considered part of it together with the segment C_U_C_S_, which, due to the particular motion of these two points, can only perform a spherical motion with the same SMC as the other five mobile links. Moreover, the U joint forbids the rotation of the distal link around axes that do not lie on the plane that passes through C_U_ and is parallel to its R-pair axes. Therefore, provided that point C_S_ does not lie on this plane, which is a particular geometric condition that can be avoided by suitably sizing the links (i.e., it is a structural singularity), the distal link cannot rotate around the segment C_U_C_S_. Since such a rotation is the only motion that would make the distal link perform a non-spherical motion around the same SMC as the other links, the conclusion is that even the distal link must perform such a spherical motion. *Q.E.D.* □

The thusly deduced spherical RRU-RRS is also obtainable from the spherical non-overconstrained 3RRS architecture proposed in [43], which was used in [8] to obtain a quasi-spherical neck brace, by eliminating one RRS limb and, successively, replacing one S pair with one U joint in one out of the two remaining RRS limbs. The 3RRS neck brace proposed in [8] has been employed for measurements [44] and rehabilitation therapies [45,46,47]. It has a wide workspace that covers 63% of the flexion/extension ROM, 65% of the axial rotation ROM and 85% of the lateral-bending ROM. Therefore, the structural simplification introduced with the novel RRU-RRS architecture is expected to extend this workspace to meet the design requirements of Table 3 and to better satisfy all the needs of those measurements and therapies where the 3RRS neck brace is employed.

### 2.3. Position Analysis of the RRU-RRS Mechanism

In the proposed RRU-RRS neck brace, for all the applications where the brace moves the patient’s head, while remaining fixed to the distal link, the actuated joints are the two R pairs of the RRS limb and the R pair of the RRU limb that is adjacent to the frame, which is fixed to the patient’s shoulders. This choice satisfies the requirement of having two actuated joints on the frame and the third one near the frame.

The position analysis of a mechanism consists of the solution of two problems: the Inverse Position Analysis (IPA) and the Forward Position Analysis (FPA). The IPA is the determination of the actuated joints’ variables for an assigned pose of the output link, which here is the distal link. Conversely, the FPA is the determination of the output link’s pose(s) compatible with assigned values of the actuated joints’ variables. In order to design a control system for the mechanism’s motion, the solution of both these problems is necessary.

**Figure 3:**
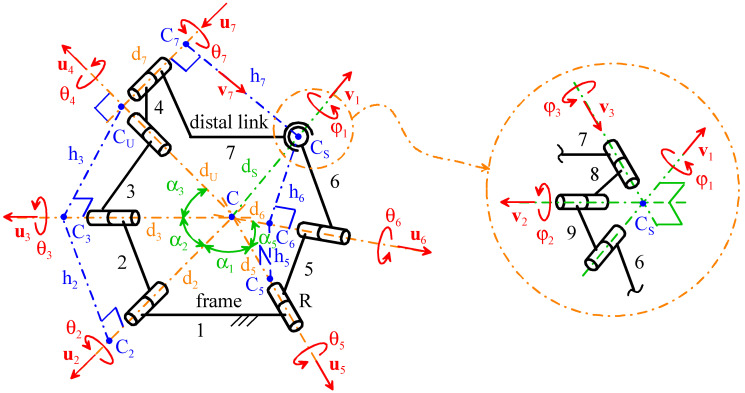
Notations.

Figure 3 shows the notations adopted here. With reference to Figure 3, the vectors **u***_i_* and the angles θ*_i_*, for *i* = 2, …, 7, are the axes’ unit vectors and the joints’ variables, respectively, for the six R pairs, and their right subscript *i* is the number associated with the R pair they refer to. The angle θ*_i_*, for *i* = 2, …, 7, is positive if counterclockwise with respect to **u***_i_*, and is equal to zero when the two links joined by the *i*-th pair are folded onto one another with the geometric elements of their end joints (i.e., the center for the S pair and the axis for the R pair) lying on the same plane. Points C, C_U_, and C_S_ are the centers of the mechanism’s spherical motion (i.e., the SMC), the U joint and the S pair, respectively. Points C_6_ and C_7_ are the feet of the perpendiculars from point C_S_ to the axes of R pairs 6 and 7, respectively. Points C_3_, C_2_, and C_5_ are the feet of the perpendiculars from points C_U_, C_3_, and C_6_, respectively, to the axes of R pairs 3, 2, and 5, respectively. *d_2_*, *d_3_*, *d_5_*, *d_6_*, *d_U_*, and *d_S_* are the distances of points C_2_, C_3_, C_5_, C_6_, C_U_, and C_S_, respectively, from point C; whereas, *d_7_* is a signed distance defined such that the vector relationship (C_U_ − C_7_) = *d_7_* **u***_7_* holds. *h_2_*, *h_3_*, *h_5_*, *h_6_*, and *h_7_* are the lengths of the segments C_2_C_3_, C_3_C_U_, C_5_C_6_, C_6_C_S_, and C_7_C_S_, respectively, The vectors **v***_1_* and **v***_7_* are unit vectors defined such that the vector relationships (C_S_ − C) = *d_S_* **v***_1_* and (C_S_ − C_7_) = *h_7_* **v***_7_* hold. φ*_i_* and v*_i_*, for *i* = 1, 2, 3, are the joint variables and the axial unit vectors, respectively, of a virtual RRR spherical kinematic chain that replaces the S pair to analytically model its kinematics. Finally, α_1_, α_2_, α_3_, and α_5_ are the angles formed by the axes of the two R pairs at the ends of links 1, 2, 3, and 5, respectively.

Using the notation defined above, the actuated joints’ variables are θ_2_, θ_5_, and θ_6_, and the following relationships among geometric constants hold:

(2a) $$d_{3}=d_{U}\cos\alpha_{3};\;h_{3}=d_{U}\sin\alpha_{3};\;d_{2}=d_{3}\cos\alpha_{2}=d_{U}\cos\alpha_{3}\cos\alpha_{2};$$

(2b) $$h_{2}=d_{2}\sin\alpha_{2}=d_{U}\cos\alpha_{3}\cos\alpha_{2}\sin\alpha_{2};\;d_{6}=\sqrt{d_{S}^{2}-h_{6}^{2}};\;\left\|C_{S}-C_{U}\right\|=\sqrt{d_{7}^{2}+h_{7}^{2}};$$

(2c) $$d_{5}=d_{6}\cos\alpha_{5}=\sqrt{d_{S}^{2}-h_{6}^{2}}\cos\alpha_{5};\;h_{5}=d_{5}\sin\alpha_{5}=\sqrt{d_{S}^{2}-h_{6}^{2}}\cos\alpha_{5}\sin\alpha_{5}$$

Equation (2a–c) and Figure 3 highlight that the independent geometric constants that fully define the mechanism geometry are only 9; that is, the constant angles α_1_, α_2_, α_3_, and α_5_ plus the linear constants *d_U_*, *d_S_*, *d_7_*, *h_6_*, and *h_7_*, which allow for the computation of all the remaining constants through Formula (2a–c).

Moreover, the following relationships hold (Figure 3):

(3a) $$(C_{2}-C)=d_{2}u_{2};\;(C_{3}-C_{2})=\frac{h_{2}}{\sin\alpha_{1}}[\sin\theta_{2}u_{2}\times u_{5}+\cos\theta_{2}(u_{2}\times u_{5})\times u_{2}]$$

(3b) $$u_{3}=u_{2}\cos\alpha_{2}+\frac{\sin\alpha_{2}}{\sin\alpha_{1}}[\sin\theta_{2}u_{2}\times u_{5}+\cos\theta_{2}(u_{2}\times u_{5})\times u_{2}]$$

(3c) $$\left(C_{U}-C_{3})=\frac{h_{3}}{\sin\alpha_{2}}[\sin\theta_{3}u_{3}\times u_{2}+\cos\theta_{3}(u_{3}\times u_{2})\times u_{3}\right]$$

(3d) $$u_{4}=u_{3}\cos\alpha_{3}+\frac{\sin\alpha_{3}}{\sin\alpha_{2}}[\sin\theta_{3}u_{3}\times u_{2}+\cos\theta_{3}(u_{3}\times u_{2})\times u_{3}]=\frac{\left(C_{U}-C\right)}{d_{U}}$$

(3e) $$u_{7}=\frac{1}{\sin\alpha_{3}}[\sin\theta_{4}u_{4}\times u_{3}+\cos\theta_{4}(u_{4}\times u_{3})\times u_{4}]$$

(3f) $$v_{7}=\sin\theta_{7}u_{4}\times u_{7}-\cos\theta_{7}u_{4};\;(C_{S}-C_{U})=h_{7}v_{7}-d_{7}u_{7}$$

(3g) $$(C_{5}-C)=d_{5}u_{5};\;(C_{6}-C_{5})=\frac{h_{5}}{\sin\alpha_{1}}[\sin\theta_{5}u_{5}\times u_{2}+\cos\theta_{5}(u_{5}\times u_{2})\times u_{5}]$$

(3h) $$u_{6}=u_{5}\cos\alpha_{5}+\frac{\sin\alpha_{5}}{\sin\alpha_{1}}[\sin\theta_{5}u_{5}\times u_{2}+\cos\theta_{5}(u_{5}\times u_{2})\times u_{5}]$$

(3i) $$\left(C_{S}-C_{6})=\frac{h_{6}}{\sin\alpha_{5}}[\sin\theta_{6}u_{6}\times u_{5}+\cos\theta_{6}(u_{6}\times u_{5})\times u_{6}\right]$$

(3j) $$v_{1}=\frac{d_{6}}{d_{S}}u_{6}+\frac{h_{6}}{d_{S}\sin\alpha_{5}}[\sin\theta_{6}u_{6}\times u_{5}+\cos\theta_{6}(u_{6}\times u_{5})\times u_{6}]=\frac{\left(C_{S}-C\right)}{d_{S}}$$

(3k) $$v_{1}\cdot u_{4}=\frac{d_{S}^{2}+d_{U}^{2}-d_{7}^{2}-h_{7}^{2}}{2d_{S}d_{U}}$$

where the three vectors $u_{2}$, $\frac{u_{2}\times u_{5}}{\sin\alpha_{1}}$, and $\frac{(u_{2}\times u_{5})\times u_{2}}{\sin\alpha_{1}}$ (the three vectors $u_{7}$, $v_{7}$, and $u_{7}\times v_{7}$) constitute a right-handed triplet of unit vectors fixed to the frame (to the distal link).

The vector closure equation of the RRU-RRS single-loop mechanism can be written as follows (see Figure 3):

(4) $$\left(C_{S}-C_{U})+(C_{U}-C_{3})+(C_{3}-C_{2})+(C_{2}-C)=(C_{S}-C_{6})+(C_{6}-C_{5})+(C_{5}-C\right)$$

The dot products of Equation (4) by $u_{2}$, $u_{2}\times u_{5}$, and $(u_{2}\times u_{5})\times u_{2}$ yield the following system of three scalar equations:

(5) $$\left\{\begin{array}{l} {}[(C_{S}-C_{U})+{\left(C_{U}-C\right)}_{RRU}]\cdot \;u_{2}={\left(C_{S}-C\right)}_{RRS}\cdot \;u_{2}\\ \left[(C_{S}-C_{U})+{\left(C_{U}-C\right)}_{RRU}]\cdot \;(u_{2}\times u_{5})={\left(C_{S}-C\right)}_{RRS}\cdot \;(u_{2}\times u_{5}\right)\\ \left[(C_{S}-C_{U})+{\left(C_{U}-C\right)}_{RRU}]\cdot \;(u_{2}\times u_{5})\times u_{2}={\left(C_{S}-C\right)}_{RRS}\cdot \;[(u_{2}\times u_{5})\times u_{2}\right] \end{array}\right.$$

where

(6a) $${\left(C_{U}-C\right)}_{RRU}=(C_{U}-C_{3})+(C_{3}-C_{2})+(C_{2}-C)$$

(6b) $${\left(C_{S}-C\right)}_{RRS}=(C_{S}-C_{6})+(C_{6}-C_{5})+(C_{5}-C)$$

System (5), after the introduction of Formula (3a–j), relates the three actuated joints’ variables θ_2_, θ_5_, and θ_6_ to the remaining three R pairs’ variables θ_3_, θ_4_, and θ_7_, which are sufficient to compute the pose of the distal link through Formula (3e,f).

#### 2.3.1. Inverse Position Analysis (IPA)

In the studied mechanism, the IPA consists of determining the values of the three-tuple (θ_2_, θ_5_, θ_6_) compatible with one assigned pose of the distal link. If the pose of the distal link is known (see Figure 3), the positions of points C_U_ and C_S_ are known in a reference system fixed to the frame. Also, since point C and unit vectors $u_{2}$ and $u_{5}$ are fixed to the frame, unit vectors $u_{4}$ and $v_{1}$ are also known in the same reference system. As a consequence, the dot products $u_{2}\cdot u_{4}$ and $v_{1}\cdot u_{5}$ are known. Such products, by exploiting Formula (3d,j), can be written as follows:

(7a) $$u_{4}\cdot u_{2}=u_{3}\cdot u_{2}\cos\alpha_{3}+\cos\theta_{3}\frac{\sin\alpha_{3}}{\sin\alpha_{2}}[(u_{3}\times u_{2})\times u_{3}]\cdot u_{2}=\cos\alpha_{2}\cos\alpha_{3}+\cos\theta_{3}\sin\alpha_{2}\sin\alpha_{3}$$

(7b) $$v_{1}\cdot u_{5}=\frac{d_{6}}{d_{S}}u_{6}\cdot u_{5}+\frac{h_{6}}{d_{S}\sin\alpha_{5}}\cos\theta_{6}[(u_{6}\times u_{5})\times u_{6}]\cdot u_{5}=\frac{d_{6}}{d_{S}}\cos\alpha_{5}+\cos\theta_{6}\frac{h_{6}\sin\alpha_{5}}{d_{S}}$$

where the following identities have been used (see Figure 3):

(8a) $$u_{3}\cdot u_{2}=\cos\alpha_{2};\;[(u_{3}\times u_{2})\times u_{3}]\cdot u_{2}=(u_{3}\times u_{2})\cdot (u_{3}\times u_{2})=\sin^{2}\alpha_{2}$$

(8b) $$u_{6}\cdot u_{5}=\cos\alpha_{5};\;[(u_{6}\times u_{5})\times u_{6}]\cdot u_{5}=(u_{6}\times u_{5})\cdot (u_{6}\times u_{5})=\sin^{2}\alpha_{5}$$

Moreover, the replacement of the explicit expressions of $u_{3}$ and $u_{6}$ given by Formulas (3b,h), respectively, into Formulas (3d,j), respectively, yields

(9a) $$u_{4}=a_{0}+a_{1}\sin\theta_{2}+a_{2}\cos\theta_{2}$$

(9b) $$v_{1}=b_{0}+b_{1}\sin\theta_{5}+b_{2}\cos\theta_{5}$$

where vectors $a_{k}$ and $b_{k}$, for *k* = 0,1,2, uniquely depend on θ_3_ and θ_6_, respectively, through the following explicit formulas:

(10a) $$a_{0}=u_{2}(\cos\alpha_{2}\cos\alpha_{3}+\sin\alpha_{2}\sin\alpha_{3}\cos\theta_{3})$$

(10b) $$a_{1}=\frac{\sin\alpha_{2}\cos\alpha_{3}}{\sin\alpha_{1}}u_{2}\times u_{5}+\frac{\sin\alpha_{3}}{\sin\alpha_{1}}[(u_{2}\times u_{5})\times u_{2}]\sin\theta_{3}-\frac{\cos\alpha_{2}\sin\alpha_{3}}{\sin\alpha_{1}}u_{2}\times u_{5}\cos\theta_{3}$$

(10c) $$a_{2}=\frac{\sin\alpha_{2}\cos\alpha_{3}}{\sin\alpha_{1}}[(u_{2}\times u_{5})\times u_{2}]+\frac{\sin\alpha_{3}}{\sin\alpha_{1}}(u_{5}\times u_{2})\sin\theta_{3}-\frac{\cos\alpha_{2}\sin\alpha_{3}}{\sin\alpha_{1}}[(u_{2}\times u_{5})\times u_{2}]\cos\theta_{3}$$

(10d) $$b_{0}=u_{5}\left(\frac{d_{6}}{d_{S}}\cos\alpha_{5}+\frac{h_{6}\sin\alpha_{5}}{d_{S}}\cos\theta_{6}\right)$$

(10e) $$b_{1}=\frac{d_{6}}{d_{S}}\frac{\sin\alpha_{5}}{\sin\alpha_{1}}u_{5}\times u_{2}+\frac{h_{6}}{d_{S}\sin\alpha_{1}}[(u_{5}\times u_{2})\times u_{5}]\sin\theta_{6}-\frac{h_{6}}{d_{S}}\frac{\cos\alpha_{5}}{\sin\alpha_{1}}(u_{5}\times u_{2})\cos\theta_{6}$$

(10f) $$b_{2}=\frac{d_{6}}{d_{S}}\frac{\sin\alpha_{5}}{\sin\alpha_{1}}[(u_{5}\times u_{2})\times u_{5}]+\frac{h_{6}}{d_{S}\sin\alpha_{1}}(u_{2}\times u_{5})\sin\theta_{6}-\frac{h_{6}}{d_{S}}\frac{\cos\alpha_{5}}{\sin\alpha_{1}}[(u_{5}\times u_{2})\times u_{5}]\cos\theta_{6}$$

The above-deduced Equations (7) and (9) allow for the closed-form solution of the IPA as below. Firstly, Equation (7a,b) yield the following values for θ_3_ and θ_6_:

(11a) $$\theta_{3}=\pm \cos^{-1}\left(\frac{u_{4}\cdot u_{2}-\cos\alpha_{2}\cos\alpha_{3}}{\sin\alpha_{2}\sin\alpha_{3}}\right)$$

(11b) $$\theta_{6}=\pm \cos^{-1}\left(\frac{d_{S}v_{1}\cdot u_{5}-d_{6}\cos\alpha_{5}}{h_{6}\sin\alpha_{5}}\right)$$

Successively, for each value of θ_3_ and θ_6_ computed through Formulas (11a,b), respectively, Equations (9a,b) allow for the computation of the corresponding value of θ_2_ and θ_5_ through the following formulas:

(12a) $$\theta_{2}=\mathrm{atan}2\left(\left[(u_{4}-a_{0})\times a_{2}]\cdot (a_{1}\times a_{2}),\;[(u_{4}-a_{0})\times a_{1}]\cdot (a_{2}\times a_{1}\right)\right)$$

(12b) $$\theta_{5}=\mathrm{atan}2\left(\left[(v_{1}-b_{0})\times b_{2}]\cdot (b_{1}\times b_{2}),\;[(v_{1}-b_{0})\times b_{1}]\cdot (b_{2}\times b_{1}\right)\right)$$

Formulas (11) and (12) lead to the computation of at most four values of the three-tuple (θ_2_, θ_5_, θ_6_). Consequently, the IPA of the studied mechanism has at most four solutions.

#### 2.3.2. Forward Position Analysis

In the studied mechanism, the FPA consists of the determination of the poses of the distal link (i.e., of unit vectors $u_{7}$ and $v_{7}$) compatible with one assigned value of the three-tuple (θ_2_, θ_5_, θ_6_). If the angles θ_2_, θ_5_, and θ_6_ are known (see Figure 3), the positions of point C_S_ and unit vectors $u_{3}$ and $u_{6}$ are known in a reference system fixed to the frame. Also, since point C and unit vectors $u_{2}$ and $u_{5}$ are fixed to the frame, unit vector $v_{1}$ is also known in the same reference system.

Consequently, Formulas (3d,k) make it possible to write the following relationship:

(13) $$\frac{d_{S}^{2}+d_{U}^{2}-d_{7}^{2}-h_{7}^{2}}{2d_{S}d_{U}}=v_{1}\cdot u_{3}\cos\alpha_{3}+\frac{\sin\alpha_{3}}{\sin\alpha_{2}}\{v_{1}\cdot (u_{3}\times u_{2})\sin\theta_{3}+v_{1}\cdot [(u_{3}\times u_{2})\times u_{3}]\cos\theta_{3}\}$$

which can be rewritten as follows:

(14) $$e_{2}\cos\theta_{3}+e_{1}\sin\theta_{3}+e_{0}=0$$

where

(15a) $$e_{2}=\frac{\sin\alpha_{3}}{\sin\alpha_{2}}v_{1}\cdot [(u_{3}\times u_{2})\times u_{3}];\;\;\;e_{1}=\frac{\sin\alpha_{3}}{\sin\alpha_{2}}v_{1}\cdot (u_{3}\times u_{2})$$

(15b) $$e_{0}=v_{1}\cdot u_{3}\cos\alpha_{3}-\frac{d_{S}^{2}+d_{U}^{2}-d_{7}^{2}-h_{7}^{2}}{2d_{S}d_{U}}$$

Equation (14) is transformable into a quadratic equation in *t* = tan(θ_3_/2) through the half-angle tangent substitution—that is, the introduction of the trigonometric identities cosθ_3_ = (1 − t^2^)/(1 + t^2^) and sinθ_3_ = 2t/(1 + t^2^)—and this is then solvable in closed form through the formula

(16) $$t_{i}=\frac{-e_{1}+{\left(-1\right)}^{i}\sqrt{e_{1}^{2}+e_{2}^{2}-e_{0}^{2}}}{\left(e_{0}-e_{2}\right)}\;\;\;i=1,2$$

which gives up to two values of θ_3_—that is, θ_3,*i*_ = 2 arctan(*t_i_*) for *i* = 1, 2—and, through Formula (3d), as many values of $u_{4}$ and corresponding positions of point C_U_.

Since the position of point C_S_ is known, for each computed position of point C_U_, a corresponding vector (C_S_−C_U_) is computable and, by introducing expression (3e) of unit vector $u_{4}$ into Formula (3f), can be used to write the following vector equation:

(17) $$(C_{S}-C_{U})=(h_{7}\sin\theta_{4}\sin\theta_{7}+d_{7}\cos\theta_{4})\frac{u_{4}\times (u_{4}\times u_{3})}{\sin\alpha_{3}}+(h_{7}\cos\theta_{4}\sin\theta_{7}-d_{7}\sin\theta_{4})\frac{\left(u_{4}\times u_{3}\right)}{\sin\alpha_{3}}-h_{7}\cos\theta_{7}u_{4}$$

where the unit vectors $u_{4}$, $\frac{\left(u_{4}\times u_{3}\right)}{\sin\alpha_{3}}$, and $\frac{u_{4}\times (u_{4}\times u_{3})}{\sin\alpha_{3}}$ constitute a right-handed triplet of unit vectors fixed to link 3 (see Figure 3). Equation (17) can be rewritten as follows:

(18) $$(C_{S}-C_{U})=g_{1}\sin\theta_{4}+g_{2}\cos\theta_{4}-h_{7}\cos\theta_{7}u_{4}$$

where $g_{1}$ and $g_{2}$ depend only on θ_7_ through the formulas

(19a) $$g_{1}=h_{7}\sin\theta_{7}\frac{u_{4}\times (u_{4}\times u_{3})}{\sin\alpha_{3}}-d_{7}\frac{\left(u_{4}\times u_{3}\right)}{\sin\alpha_{3}}$$

(19b) $$g_{2}=d_{7}\frac{u_{4}\times (u_{4}\times u_{3})}{\sin\alpha_{3}}+h_{7}\sin\theta_{7}\frac{\left(u_{4}\times u_{3}\right)}{\sin\alpha_{3}}$$

By noting that the triplet of vectors $u_{4}$, $g_{1}$, and $g_{2}$ are mutually orthogonal, Equation (18) yields the following solution formulas for θ_4_ and θ_7_:

(20a) $$\theta_{7}=\pm \cos^{-1}\left(\frac{(C_{U}-C_{S})\cdot u_{4}}{h_{7}}\right)$$

(20b) $$\left.\begin{array}{l} \sin\theta_{4}=\frac{(C_{S}-C_{U})\cdot g_{1}}{g_{1}\cdot g_{1}}\\ \cos\theta_{4}=\frac{(C_{S}-C_{U})\cdot g_{2}}{g_{2}\cdot g_{2}} \end{array}\right\}\Rightarrow \theta_{4}=\mathrm{atan}2\left[(C_{S}-C_{U})\cdot g_{1},\;(C_{S}-C_{U})\cdot g_{2}\right]$$

The conclusion is that the FPA leads to computing at most four poses of the distal link compatible with one set of values of the actuated joint variables (i.e., of (θ_2_, θ_5_, θ_6_)). Indeed, Equation (16) gives two values for θ_3_, which, when replaced into Equation (3d), yield as many values for $u_{4}$ and $\left(C_{U}-C_{S}\right)$, whose introduction into Equation (20a) gives at most four values for θ_7_, to which Equation (20b) associates as many values of θ_4_. All the FPA solutions are computable through closed-form formulas.

### 2.4. Instantaneous Kinematics and Singularity Analysis of the RRU-RRS Mechanism

The velocity, $\dot{C}$, of point *C*, when considered fixed to the distal link, and the angular velocity, **ω**, of the distal link are computable by moving from the frame to the distal link along one or the other of the two limbs. This computation yields the following two different analytic expressions for **ω** and $\dot{C}$ (see Figure 3):

(21a) $$RRS\;limb\Rightarrow \left\{\begin{array}{l} {\dot{C}}_{S}=({\dot{\theta }}_{5}u_{5}+{\dot{\theta }}_{6}u_{6})\times (C_{S}-C)\\ \dot{C}={\dot{C}}_{S}+\omega \times (C-C_{S})\\ \omega ={\dot{\theta }}_{5}u_{5}+{\dot{\theta }}_{6}u_{6}+{\dot{\varphi }}_{1}v_{1}+{\dot{\varphi }}_{2}v_{2}+{\dot{\varphi }}_{3}v_{3}\\ (C_{S}-C)=d_{S}v_{1} \end{array}\rangle \Rightarrow \dot{C}=({\dot{\varphi }}_{1}v_{1}+{\dot{\varphi }}_{2}v_{2}+{\dot{\varphi }}_{3}v_{3})\times (C-C_{S})={\dot{\varphi }}_{2}d_{S}v_{2}\times v_{1}+{\dot{\varphi }}_{3}d_{S}v_{3}\times v_{1}\right.$$

(21b) $$RRU\;limb\Rightarrow \left\{\begin{array}{l} {\dot{C}}_{U}=({\dot{\theta }}_{2}u_{2}+{\dot{\theta }}_{3}u_{3})\times (C_{U}-C)\\ \dot{C}={\dot{C}}_{U}+\omega \times (C-C_{U})\\ \omega ={\dot{\theta }}_{2}u_{2}+{\dot{\theta }}_{3}u_{3}+{\dot{\theta }}_{4}u_{4}+{\dot{\theta }}_{7}u_{7}\\ (C_{U}-C)=d_{U}u_{4} \end{array}\rangle \Rightarrow \dot{C}=({\dot{\theta }}_{4}u_{4}+{\dot{\theta }}_{7}u_{7})\times (C-C_{U})=-{\dot{\theta }}_{7}d_{U}u_{7}\times u_{4}\right.$$

Equating the so-obtained analytic expressions of **ω** and $\dot{C}$ yields the following velocity-loop equations of the studied mechanism:

(22a) $$\dot{C}=\dot{C}\;\Rightarrow \;{\dot{\varphi }}_{2}d_{S}v_{2}\times v_{1}+{\dot{\varphi }}_{3}d_{S}v_{3}\times v_{1}+{\dot{\theta }}_{7}d_{U}u_{7}\times u_{4}=0$$

(22b) $$\omega =\omega \;\Rightarrow \;{\dot{\theta }}_{5}u_{5}+{\dot{\theta }}_{6}u_{6}+{\dot{\varphi }}_{1}v_{1}+{\dot{\varphi }}_{2}v_{2}+{\dot{\varphi }}_{3}v_{3}={\dot{\theta }}_{2}u_{2}+{\dot{\theta }}_{3}u_{3}+{\dot{\theta }}_{4}u_{4}+{\dot{\theta }}_{7}u_{7}$$

Equation (22a) is a linear and homogeneous system of three equations in three unknowns that can be written in matrix form as follows:

(23) M ζ=0

where the following definitions for the coefficient matrix **M** and the unknown vector **ζ** have been introduced

(24) $$M=[d_{S}v_{2}\times v_{1},\;d_{S}v_{3}\times v_{1},\;d_{U}u_{7}\times u_{4}];\;\;\;\zeta =\left(\begin{array}{c} {\dot{\varphi }}_{2}\\ {\dot{\varphi }}_{3}\\ {\dot{\theta }}_{7} \end{array}\right)$$

If det(**M**) ≠ 0, system (23) admits only the trivial solution **ζ** = **0**, which corresponds to $\dot{C}=0$ (see Equations (21a) and (21b)) and makes the distal link perform a spherical motion with point *C* as its SMC. Conversely, if det(**M**) = 0, system (23) admits an infinity of solutions for **ζ**, which can also be non-null; that is, a *constraint singularity* [48] occurs where the distal link can exit from the spherical motion. From an analytic point of view, the determinant of a 3 × 3 matrix is the mixed product of its column vectors, which, in this case, yields the following formula:

(25) $$\det(M)=d_{U}d_{S}^{2}(u_{7}\times u_{4})\cdot [(v_{2}\times v_{1})\times (v_{3}\times v_{1})]$$

By excluding the case in which one of the three vectors is a null vector, a mixed product is equal to zero if and only if the three involved vectors are all parallel to one plane. In the case under study (see Figure 3), none of the three involved vectors is a null vector. The two vectors $v_{2}\times v_{1}$ and $v_{3}\times v_{1}$ are both perpendicular to $v_{1}$ and identify a pencil of parallel planes that are perpendicular to the line passing through points *C* and *C_S_*; whereas, the remaining third vector $u_{7}\times u_{4}$ is perpendicular to the plane of the U joint’s cross link, which is the one located by the three points *C*, *C_7_*, and *C_U_* (Figure 3). Consequently, the parallelism of these three vectors to one plane (i.e., a constraint singularity) can occur if and only if point *C_S_* lies on the plane of the U joint’s cross link. Such a result analytically confirms what was concluded, using kinematic reasoning, in Section 2.2 when proving the statement reported there. This geometric condition is avoidable by sizing the mechanism such that the triangle C_U_CC_S_ does not degenerate into a segment; that is, by imposing

(26) $$\bar{CC_{U}}+\bar{CC_{S}}>\bar{C_{U}C_{S}}\Rightarrow d_{U}+d_{S}>\sqrt{d_{7}^{\;2}+h_{7}^{\;2}}$$

during the design of the mechanism, and by assembling the mechanism at a configuration where point C_S_ does not lie on the plane of the U-joint’s cross link. Indeed, if the mechanism is assembled at a non-singular configuration—that is, with point C_S_ out of the plane of the U-joint’s cross link—the distal link can only perform spherical motions that make the triangle C_7_C_U_C_S_ rigidly rotate around the line passing through points *C* and *C_U_*, which always keeps point C_S_ out of the plane of the U-joint’s cross link.

Condition (26) contains only geometric constants of the mechanism (i.e., it is a *structural* condition). Therefore, if the mechanism is sized such that it satisfies inequality (26) and is assembled with point C_S_ out of the plane of the U-joint’s cross link, the possible occurrence of a constraint singularity is definitely excluded and the distal link can only perform spherical motion. This implies that **ζ** is always a null vector, which leads to simplification of Equation (22b) as follows:

(27) $$A\;\dot{x}=B\;\dot{q}$$

where the following definitions have been introduced:

(28) $$A=[u_{3},\;u_{4},\;-v_{1}];\;\;\;\dot{x}=\left(\begin{array}{c} {\dot{\theta }}_{3}\\ {\dot{\theta }}_{4}\\ {\dot{\varphi }}_{1} \end{array}\right);\;\;\;B=[u_{5},\;u_{6},\;-u_{2}];\;\;\;\dot{q}=\left(\begin{array}{c} {\dot{\theta }}_{5}\\ {\dot{\theta }}_{6}\\ {\dot{\theta }}_{2} \end{array}\right)$$

Formulas (28) highlight that **A** and **B** are 3 × 3 matrices that depend on the mechanism configuration. Such matrices are named *Jacobians*.

Equation (27) relates the actuated joint rates (i.e., ${\dot{\theta }}_{2}$, ${\dot{\theta }}_{5}$, and ${\dot{\theta }}_{6}$), which are the instantaneous inputs, to the remaining R-pair joint rates (i.e., ${\dot{\theta }}_{3}$ and ${\dot{\theta }}_{4}$ (see Equation 21b)) that are necessary to compute the angular velocity, **ω**, which is the instantaneous output, and is sufficient to identify the instantaneous spherical motion of the distal link. This is the *input-output instantaneous relationship* of the studied mechanism. Equation (27) allows for the solution of two problems [49,50,51]: the Inverse Instantaneous Kinematics (IIK) problem and the Forward Instantaneous Kinematics (FIK) problem. The IIK problem is the determination of the instantaneous inputs’ values (i.e., in the studied case, of ${\dot{\theta }}_{2}$, ${\dot{\theta }}_{5}$, and ${\dot{\theta }}_{6}$) compatible with one assigned set of instantaneous outputs (i.e., in the studied case, of **ω** or, which is the same, of ${\dot{\theta }}_{3}$ and ${\dot{\theta }}_{4}$). Vice versa, the FIK problem is the determination of the instantaneous outputs’ values (i.e., in the studied case, of **ω** or, which is the same, of ${\dot{\theta }}_{3}$ and ${\dot{\theta }}_{4}$) compatible with one assigned set of instantaneous inputs (i.e., in the studied case, of ${\dot{\theta }}_{2}$, ${\dot{\theta }}_{5}$, and ${\dot{\theta }}_{6}$).

The IIK and FIK solutions depend on the mechanism’s configuration since matrices **A** and **B** depend on it. The mechanism configurations that make Instantaneous Kinematics (IK) problems indeterminate are the mechanism’s *singularities*. Such singularities are collectable into three main groups [49]: (i) those that make the IIK indeterminate, named serial (or type-I) singularities; (ii) those that make the FIK indeterminate, named parallel (or type-II) singularities; and (iii) those that make both the IK problems indeterminate, named type-III singularities.

At a serial singularity, the output link (i.e., the distal link in the studied case) can stay at rest even though the actuated joint rates are different from zero; that is, it has a local reduction in DOFs since the motion of one or more actuated joints does not affect its motion. Since this kinematic condition identifies configurations that are at the boundaries of the workspace, serial singularities are located at the workspace boundaries, and identifying them is one of the methods used for determining the output link’s workspace.

Conversely, at a parallel singularity, the output link can perform an instantaneous motion even though the actuated joints are at rest; that is, it locally acquires further DOFs since the actuated joints are no longer able to control its motion. Parallel singularities may be located inside the workspace. The virtual work principle demonstrates that, at a parallel singularity, even an infinitesimal external load applied to the output link requires that one or more actuators must apply an infinite generalized torque to equilibrate it. Such a static condition leads to the breakdown either of some actuators or of some links. Therefore, parallel singularities must be identified during the design stage and avoided during operation.

The analysis of Equation (27) reveals that serial singularities satisfy the analytic and geometric condition

(29) $$\det(B)=-\;(u_{2}\times u_{5})\cdot \;u_{6}=0$$

and that parallel singularities satisfy this other analytic and geometric condition:

(30) $$\det(A)=-\;(u_{3}\times u_{4})\cdot \;v_{1}=0$$

Condition (29) (Condition (30)) is satisfied when the involved unit vectors are coplanar.

The configurations farthest from parallel singularities are those that provide the best kinetostatic performances of a parallel mechanism. In the case under study, these configurations are those that maximize the absolute value of det(**A**) (see Equation (30)). From a geometric point of view, the identity $(u_{3}\times u_{4})\times (u_{4}\times v_{1})=[(u_{3}\times u_{4})\cdot \;v_{1}]u_{4}$, which implies $\left\|\left(u_{3}\times u_{4})\times (u_{4}\times v_{1}\right)\right\|=\left\|(u_{3}\times u_{4})\cdot \;v_{1}\right\|$, leads to the conclusion that they occur when the plane of the triangle C_S_CC_U_ is perpendicular to the plane of the triangle C_3_CC_U_ (see Figure 3). Consequently, the angle μ between these two planes plays the role of a *transmission angle* for this mechanism and the parameter $s_{\mu }=\sin\mu =\frac{\left\|\left(u_{3}\times u_{4})\times (u_{4}\times v_{1}\right)\right\|}{\left\|\left(u_{3}\times u_{4}\right)\right\|\left\|\left(u_{4}\times v_{1}\right)\right\|}$, which is dimensionless and equal to 1 (equal to 0) for μ = 90° (for μ = 0 or μ = 180°; that is, at a parallel singularity), is adoptable as a performance index. As a rule of thumb, for this type of application, which does not require high precision and does not involve high loads (it is worth stressing that, when the brace guides the head motion, the head weight still is carried by the neck), a maximum deviation of ±70° from 90° is still acceptable for μ. Thus, in the studied case, the minimum permissible value for s_μ_ is 0.342.

## 3. Results

In this section, the above-reported kinematic analyses are used to size an RRU-RRS spherical mechanism that matches the design requirements of dynamic neck braces reported in Table 3. As stressed in the previous section, the independent geometric constants that fully define the geometry of this mechanism are nine (Figure 3): the four angles α_1_, α_2_, α_3_, and α_5_, plus the five lengths *d_U_*, *d_S_*, *d_7_*, *h_6_*, and *h_7_*. Consequently, the dimensional synthesis problem to address is the determination of the values of these nine constants that make the free-from-singularity orientation workspace of the distal link match the design requirements of Table 3.

Since the studied mechanism is spherical, the distal link’s orientation workspace remains unchanged when the mechanism is scaled. Scalability is a sought-after feature when designing an orthosis since it must be adaptable to the size of the patient without changing its mobility. In addition, it makes it possible to normalize the linear constants that must be sized. Accordingly, in the studied case, the five constant lengths are replaced by their ratios to *d_S_*; that is, by *d_U_*/*d_S_*, 1 = *d_S_*/*d_S_*, *d_7_*/*d_S_*, *h_6_*/*d_S_* and *h_7_*/*d_S_*. Therefore, the independent constants to be determined are reduce to eight: the four angles α_1_, α_2_, α_3_, and α_5_, plus the four dimensionless ratios *d_U_*/*d_S_*, *d_7_*/*d_S_*, *h_6_*/*d_S_*, and *h_7_*/*d_S_*.

With reference to Figure 1b and Figure 3, in order to identify a symmetric brace that is also easy to wear, the following assumptions are introduced:

(i) Point C is located at the centroid of vertebra C4 and is the origin of the two Cartesian reference systems (Figure 1b), one fixed to the frame (i.e., to the shoulders of the patient) and the other fixed to the distal link (i.e., to the skull of the patient), that coincide with one another at the neutral position;
(ii) In the frame (link 1), point C and the two R-pair axes fixed to the frame lie on the xy-coordinate plane of the reference system fixed to the frame with the two R-pair axes that are symmetrically located with respect to the sagittal plane (Figure 1);
(iii) In the distal link, the vectors **u**_7_, **v**_7_, and $\left(C_{U}-C_{S}\right)$ are all parallel to the xy-coordinate plane of the reference system fixed to the distal link with the points *C_U_* and *C_S_* that lie on the yz-coordinate plane of the same reference system;
(iv) α_1_ = 60°, α_2_ = α_5_, sinα_3_ = *h_6_*/*d_S_*, 0 = *d_7_*/*d_S_*, 1 = *d_U_*/*d_S_*, and **v**_1_·**u**_4_ = cosα_1_.

The assumption 0 = *d_7_*/*d_S_* leads to $\bar{C_{U}C_{S}}=h_{7}$ (Figure 3), which, when combined with the assumptions α_1_ = 60°, **v**_1_·**u**_4_ = cosα_1,_ and 1 = *d_U_*/*d_S_*, leads to the conclusion that *h_7_*/*d_S_* = 1; that is, the triangle CC_U_C_S_ is equilateral. Such a triangle, for the assumptions (ii) and (iii), is located on the yz-coordinate plane of the distal link and, at the neutral position, is perpendicular to the transverse (horizontal) plane (Figure 1).

**Figure 4:**
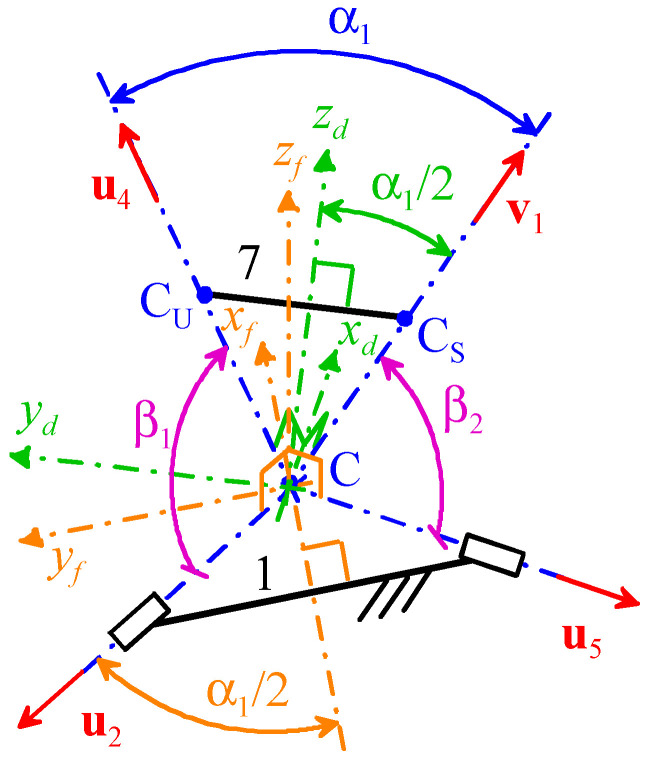
Frame (link 1) and distal link (link 7) in a generic configuration together with the Cartesian reference systems *Cx_f_y_f_z_f_*, fixed to the frame, and *Cx_d_y_d_z_d_*, fixed to the distal link.

Figure 4, shows the frame (link 1) and the distal link (link 7) in a generic configuration together with the Cartesian reference systems *Cx_f_y_f_z_f_*, fixed to the frame, and *Cx_d_y_d_z_d_*, fixed to the distal link, for a mechanism sized in line with the above-listed assumptions. Hereafter, **i***_f_*, **j***_f_*, and **k***_f_* (**i***_d_*, **j***_d_* and **k***_d_*) will denote the unit vectors, respectively, of the x, y, and z coordinate axes of *Cx_f_y_f_z_f_* (of *Cx_d_y_d_z_d_*). All of the above-listed assumptions leave only the geometric constants α_2_ (= α_5_) and α_3_ (= arcsin(*h_6_*/*d_S_*)) to be determined by imposing the design requirements from Table 3, and lead to compute the following values of vector components:

(31a) u2f=−cosα12sinα120=−320.50;   u5f=−cosα12−sinα120=−32−0.50;

(31b) u4d=0sinα12cosα12=00.532;   v1d=0−sinα12cosα12=0−0.532;

(31c) u7d=−100;   v7d=0−10;

where the left superscripts *f* or *d* on vector symbols denote vectors measured in the reference system fixed to the frame or to the distal link, respectively.

The rotation matrix, *^f^***R***_d_*, that transforms a vector, *^d^***a**, measured in *Cx_d_y_d_z_d_* to the same vector, *^f^***a**, measured in *Cx_f_y_f_z_f_* through the relationship *^f^***a =** *^f^***R***_d_ ^d^***a** has the general form

(32) Rdf=idfjdfkdf

which, by introducing the Cardan angles ψ_1_, ψ_2_, and ψ_3_ of the ZYX convention, yields the following explicit formula:

(33) Rdf=cosψ1cosψ2cosψ1sinψ2sinψ3−sinψ1cosψ3cosψ1sinψ2cosψ3+sinψ1sinψ3sinψ1cosψ2sinψ1sinψ2sinψ3+cosψ1cosψ3sinψ1sinψ2cosψ3−cosψ1sinψ3−sinψ2cosψ2sinψ3cosψ2cosψ3

It is easy to realize that the angles ψ_1_, ψ_2,_ and ψ_3_ defined in this way correspond, respectively, to the axial rotation, the flexion/extension, and the lateral bending of the head (Figure 1b). With reference to Figure 3 and Figure 4, the Orientation Workspace (OW) of the distal link can be defined as the set of all the rotation matrices, *^f^***R***_d_*, that identify distal link orientations for which β_1_, β_2_ ≤ α_2_ + α_3_, in formulas

(34) OW≡Rdf  β1,β2≤α2+α3

Since only the sum of α_2_ and α_3_ enters into Formula (34), without losing generality, the further assumption α_2_ = α_3_ can be adopted, which makes the limbs more symmetric and reduces the dimensional synthesis to the determination of only one angle. Also, since, in Formula (34), β_1_ and β_2_ are the lower extreme of the admissible range of values for α_2_ + α_3_, the dimensional synthesis can be implemented by looking for the maximum values β_1,max_ and β_2,max_ of β_1_ and β_2_, respectively, that are necessary to satisfy all the design requirements of Table 3 and, then, by choosing α_2_ = α_3_ > max(β_1,max_, β_2,max_)/2. From an analytic point of view (Figure 4), the following relationships hold:

(35a) cosβ1=u2⋅u4=u2Tf Rdf u4d⇒β1=cos−1u2Tf Rdf u4d

(35b) cosβ2=u5⋅v1=u5Tf Rdf v1d⇒β2=cos−1u5Tf Rdf v1d

The introduction of the design requirements of Table 3 into Formula (33) and of the resulting rotation matrix *^f^***R***_d_* together with the vector data given by Equation (31) into Formula (35), yields the extreme values of β_1_ and β_2_ reported in Table 4. Analysis of Table 4 reveals that the condition α_2_ = α_3_ > max(β_1,max_, β_2,max_)/2 is satisfied by choosing α_2_ = α_3_ = 56°. Table 5 summarizes the values of the geometric constants of the RRU-RRS neck brace sized in this way, which satisfy all the design requirements of Table 3.

**Figure 5:**
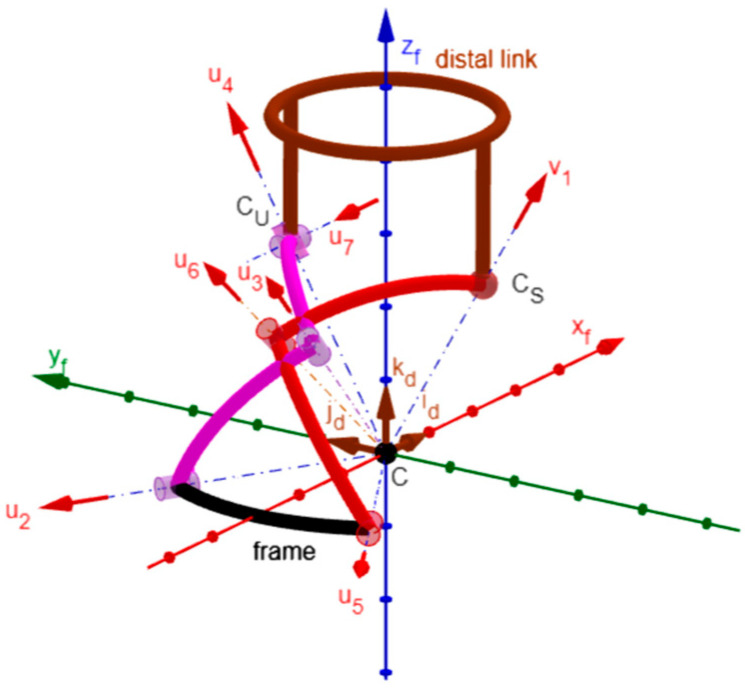
Three-dimensional kinematic model of the neck brace with sizes drawn from Table 5 and the distal link at the neutral position (the circular ring of the distal link is the harness that fixes it to the patient’s forehead).

**Figure 6:**
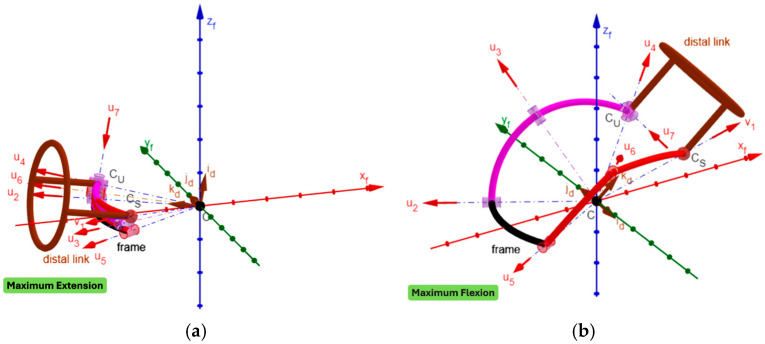
Three-dimensional kinematic model of the neck brace with sizes drawn from Table 5 and the distal link at the maximum extension (**a**) and at the maximum flexion (**b**).

**Figure 7:**
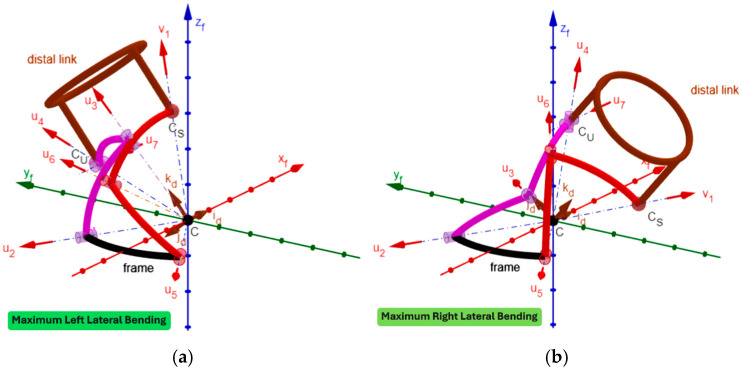
Three-dimensional kinematic model of the neck brace with sizes drawn from Table 5 and the distal link at the maximum left lateral bending (**a**) and at the maximum right lateral bending (**b**).

**Figure 8:**
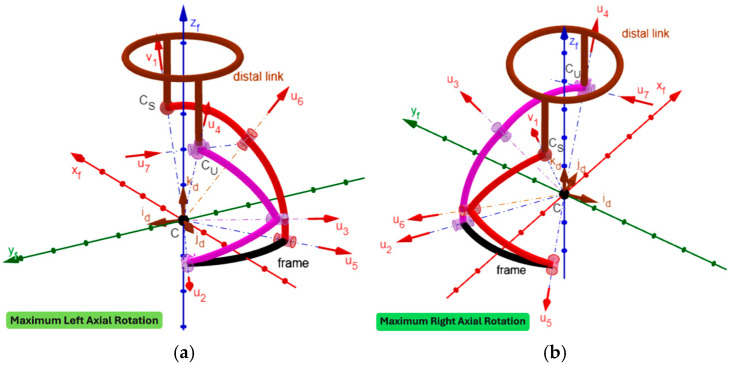
Three-dimensional kinematic model of the neck brace with sizes drawn from Table 5 and the distal link at the maximum left axial rotation (**a**) and at the maximum right axial rotation (**b**).

By using GeoGebra [52,53,54,55], an animated 3D kinematic model of the neck brace, sized with the values reported in Table 5, was built and used to check that, during motion, the singularity conditions, identified in Section 2.4, do not occur inside the workspace. Such a model controls/changes the mechanism configuration by using the inverse position–analysis relationships determined in Section 2.3.1 with the distal link orientation that is assigned by the user through the above-defined angles ψ_1_, ψ_2,_ and ψ_3_ (see Formula (33) and the accompanying comments). This control algorithm is the same as the one that a controller should use to move the real neck brace during a passive mobilization program for the patient’s neck. The built model is indeed a kinematic digital twin of the mechanism that can be used to simulate its motion. Figure 5 shows the 3D kinematic model built in this way with the distal link at the neutral position, and Figure 6, Figure 7 and Figure 8 show the mechanism configurations with the distal link at the extreme poses of flexion/extension, lateral bending, and axial rotation, respectively. In the Supplementary Materials accompanying this paper, the interested reader can find the GeoGebra program used to build the model and four videos that show the neutral position from different points of view, the axial rotation animation, the flexion/extension animation, and the lateral bending animation. These three animations clearly show that the mechanism sized in this way has no singularity inside its workspace.

Regarding the kinetostatic performance, the index s_μ_ is equal to 0.85 (corresponding to μ = 58.31°) at the neutral position. During one cycle of flexion–extension, s_μ_ assumes its minimum value, equal to 0.11 (corresponding to μ = 6.14°), at the maximum extension, and increases with the reduction in the extension, thus reaching its acceptable value (i.e., 0.342) at 55° of extension, its maximum value (i.e., 1) at 28.65° of flexion, and the value 0.82 (corresponding to μ = 54.98°) at the maximum flexion.

During one cycle of axial rotation, s_μ_ assumes its minimum value, equal to 0.6 (corresponding to μ = 36.91° and greater than its minimum acceptable value (i.e., 0.342)), at the maximum right rotation and increases with the reduction in the right rotation, thus reaching its maximum value (i.e., 1) at 33.8° of left rotation and the value 0.77 (corresponding to μ = 50.56°) at the maximum left rotation.

During one cycle of lateral bending, s_μ_ assumes its minimum value, equal to 0.58 (corresponding to μ = 35.3° and greater than its minimum acceptable value (i.e., 0.342)), at the maximum left bending, and increases with the reduction in the left bending, thus reaching the maximum value, equal to 0.98 (corresponding to μ = 77.17°) at the maximum right bending.

In short, only near the maximum extension (i.e., for extensions between 56° and 80°) s_μ_ assumes critical values. Nevertheless, since this region is near the extreme extension, the possible locking of the two passive joints, the one connecting links 2 and 3, and the other one connecting links 3 and 4, is avoidable by introducing a non-linear spring that intervenes only in this region (i.e., an elastic limit switch) to force the joint to unlock.

## 4. Discussion

The above-reported results can be summarized as follow. The proposed architecture is simple to control since its position–analysis problems are solvable in closed form. In addition, it is able to satisfy all the design requirements of a dynamic neck brace by providing a free-from-singularity workspace that covers the whole ROM of a human neck.

From a manufacturing point of view, the link interferences visible in Figure 5, Figure 6, Figure 7 and Figure 8 can be easily eliminated by making the links lie on different concentric spherical shells. Moreover, the single-loop architecture of the proposed mechanism makes it possible to mount links 3, 2, 1, 5, and 6 (see Figure 3) as a serial chain of binary links. This feature makes the intersection of all R-pair axes at the SMC (point C in Figure 3) much easier to obtain, during the mechanism assembly, than it would be in a multi-loop architecture. Finally, the fact that the proposed architecture has three DOFs when considered as a spatial mechanism (i.e., for *f* = 6 in Equation (1)) makes it work as a quasi-spherical mechanism if the intersection of all the R-pair axes at the SMC is not obtained during assembly.

When the mechanism works as a quasi-spherical mechanism (i.e., when the SMCs of the two limbs, named SMC_RRS_ and SMC_RRU_ in Section 2.2, do not coincide with one another), the formulas of the IPA solution reported in Section 2.3.1 still hold since their deduction does not use the coincidence of SMC_RRS_ and SMC_RRU_. Conversely, those of the FPA solution reported in Section 2.3.2 do not hold any longer, but the FPA still is solvable in closed form with a technique similar to the one reported in Section 2.3.2. Consequently, the proposed neck brace remains easy to use even in those applications where the quasi-spherical motion of the neck is not negligible.

How to locate the brace’s SMC (point C in Figure 2, Figure 3, Figure 4, Figure 5, Figure 6, Figure 7 and Figure 8) on the patient’s neck needs a deeper discussion. In the literature (see, for instance, [22,24,27,56,57]), the neck’s movements are analyzed by considering the three neck rotations mainly as three planar motions occurring in three mutually orthogonal planes. This approach leads to the identification of three different Instantaneous Axes of Rotation (IAR) that are mutually orthogonal and change their positions during a finite movement of the neck. Consequently, if these data were available or, as an alternative, the fixed axode [42] traced by the Instantaneous Screw Axis (ISA) during the neck motion were known, the best location of the brace’s SMC would be the point that minimizes the sum of its distances from all these axes. The determination of such a point is cumbersome, impractical, and, ultimately, unnecessary for making a patient wear a neck brace. Indeed, as stressed in Section 2.1, the cervical spine is a redundant spatial mechanism. Its redundant mobility makes it able to perform many different paths to move the distal link from one pose to another, provided that the path keeps the joints’ motion within their ROM. This condition is certainly satisfied during a spherical motion of the distal link that has its SMC nearly coincident with the centroid of vertebra C4 (Figure 1b).

**Figure 9:**
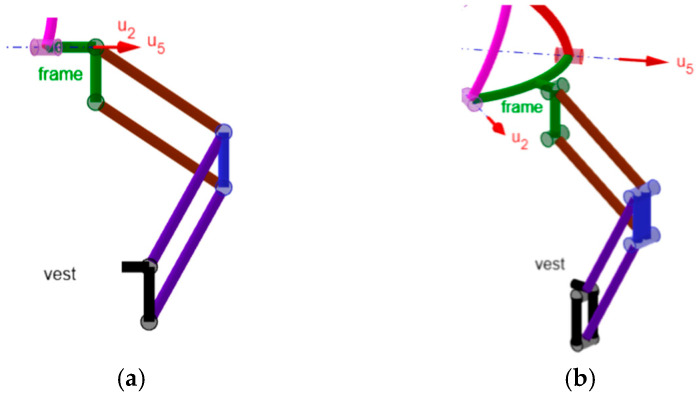
Double parallelogram mechanism mounted on the frame of the neck brace: (**a**) projection in the sagittal plane, and (**b**) 3D view.

**Figure 10:**
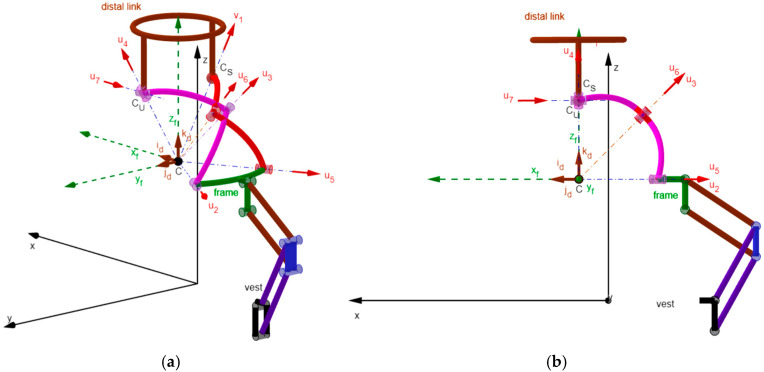
RRU-RRS spherical neck brace in the neutral position combined with a double parallelogram mechanism that moves point C in the sagittal plane: (**a**) 3D view, and (**b**) projection in the sagittal plane.

To reach this location, the neck brace must contain additional joints (alignment joints) that make the brace’s SMC (point C in Figure 3) translate in the sagittal plane by moving the brace’s frame (link 1 in Figure 3) with respect to the vest that fixes the brace to the patient’s shoulders. Of course, once the brace’s SMC has been correctly located all the alignment joints must be locked. Many planar kinematic chains can provide this two-DOF translation; among these chains, the double parallelogram mechanism (Figure 9 and Figure 10) may be the most practical since it contains only passive R pairs, which are easy to move and lock.

The identification of the centroid of vertebra C4 on the patient’s neck could be achieved through radiographs, but the use of self-alignment techniques [33,34] is more practical for the identification of the best location for the brace’s SMC. In this case, such techniques consist of the implementation of the following steps:

(i) The patient, with their shoulders and neck in the neutral position (Figure 1), wears the brace with the alignment joints unlocked and the actuated joints non-actuated (i.e., with the actuators unpowered and unlocked).
(ii) The physiotherapist makes the brace’s SMC lie on the sagittal plane (Figure 1), the axes of the two R pairs adjacent to the frame (link 1 in Figure 3) parallel to the transversal plane, and the plane of motion of the alignment joints parallel to the sagittal plane. Then, he fixes the brace’s vest to the patient shoulders and the brace’s distal link (link 7 in Figure 3) to the patient’s forehead.
(iii) The patient sequentially performs one full cycle of flexion/extension, one full cycle of axial rotation, and one full cycle of lateral bending, during which the values assumed by the joint variables of the alignment joints are recorded.
(iv) The average values of the joint variables recorded in step (iii) are computed.
(v) The physiotherapist locks the alignment joints at the values of their joint variables computed in step (iv) and then makes the patient repeat the three full-cycle motions to check that he does not feel distress or pain in the neck when moving the head and whether a ROM reduction occurs when moving the head with the alignment joints locked in place.

Regarding brace’s wearability, the spherical nature of the proposed brace makes it scalable without affecting its motion and, consequently, its motion control system. Therefore, adapting the size of the brace to the size of the patient can be simply achieved by providing either different brace sizes, or a brace with links whose dimensions are adjustable according to the size of the patient. Also, since the geometric constants that affect the brace’s motion are only the four angles α_1_, α_2_, α_3_, and α_5_, and the four dimensionless ratios *d_U_*/*d_S_*, *d_7_*/*d_S_*, *h_6_*/*d_S_,* and *h_7_*/*d_S_*, scaling the brace can be achieved by varying *d_S_* while keeping these eight parameters unchanged. This means that, for instance, the shape of the distal link can be modified freely provided that *d_7_*/*d_S_* and *h_7_*/*d_S_* are kept unchanged and that similar considerations hold for all the other links. In short, designers have many parameters available to improve the brace’s wearability and adaptability.

## 5. Conclusions

Design requirements for a dynamic neck brace were determined from the literature, and a novel type of dynamic neck brace was proposed. This is based on a three-DOF single-loop spherical mechanism that is not overconstrained.

Its kinematics and mobility were analyzed. These analyses reveal that the position analysis problems have closed-form solutions, which greatly simplify the design of the control system, and that the brace can be sized and assembled easily in a way that prevents constraint singularities.

Furthermore, a kinematic digital twin of the brace was created in GeoGebra to test its ability to cover the entire range of motion (ROM) of a human neck without passing through singular configurations. Virtual experiments conducted with this digital twin proved its ability to do so.

Finally, a simple procedure for ensuring that patients wear the brace correctly is presented. This procedure is based on self-alignment techniques and additional alignment joints.

All of the above results mean that the proposed neck brace can be used as a static and/or dynamic brace in all neck rehabilitation and healing therapies without any limitations.

## Figures and Tables

**Table 1:** Approximate ROM for the Three Planes of Movement (adapted from [21]).

Joint or Region	Flexion(+)/Extension(−) (Sagittal Plane, (°))	Axial Rotation (Horizontal Plane, (°))	Lateral Bending (Coronal Plane, (°))
O–C1 joint	+5/−10	Negligible	About ±5
C1–C2 joint	+5/−10	±35 ÷ 40	Negligible
C2–C7 region	+35 ÷ 40/−55 ÷ 60	±30 ÷ 35	±30 ÷ 35
Total	+45 ÷ 50/−75 ÷ 80	±65 ÷ 75	±35 ÷ 40

**Table 2:** Mean ROM for each intervertebral joint (adapted from [22]; the total ROMs reported in [22] have been divided by considering that axial rotation and lateral bending are symmetric with respect to the neutral position and that the flexion ROM is roughly half of the extension ROM).

Joint	Flexion(+)/Extension(−) (Sagittal Plane, (°))	Axial Rotation (Horizontal Plane, (°))	Lateral Bending (Coronal Plane, (°))
O–C1	+5/−10	Negligible	About ±1.5
C1–C2	+5/−10	±41.5	Negligible
C2–C3	+4/−8	±3	±7
C3–C4	+6/−11	±6.5	±7
C4–C5	+7/−12	±6.5	±7
C5–C6	+7/−14	±6.5	±7
C6–C7	+8/−15	±7	±7
Total	+42/−80	±71	±36.5

**Table 3:** Design requirements for the ROM of a dynamic neck brace.

	Flexion(+)/Extension(−) (Sagittal Plane, (°))	Axial Rotation (Horizontal Plane, (°))	Lateral Bending (Coronal Plane, (°))
**ROM**	+50/−80	±75	±40

**Table 4:** Maximum values of β_1_ and β_2_, computed through Equations (35a,b), which correspond to the extreme values of the ROMs reported in Table 3 (the computed values of β_1_ and β_2_ have been rounded up to degrees).

Rotation	(ψ_1_, ψ_2_, ψ_3_) (°)	β_1_ * (°)	β_2_ * (°)
Flexion	(0, 50, 0)	109	109
Extension	(0,−80, 0)	9	9
Left Axial Rotation	(75, 0, 0)	62	111
Right Axial Rotation	(−75, 0, 0)	111	62
Right Lateral Bending	(0, 0, 40)	95	62
Left Lateral Bending	(0, 0, −40)	62	95

* The values of β_1_ and β_2_ have been rounded up to degrees.

**Table 5:** Values of the geometric constants of the RRU-RRS neck brace that make it satisfy all the design requirements of Table 3.

α_1_ (°)	α_2_ (°)	α_3_ (°)	α_5_ (°)	*h_6_*/*d_S_*	*h_7_*/*d_S_*	*d_7_*/*d_S_*	*d_U_*/*d_S_*
60	56	56	56	0.829	1	0	1

## Data Availability

The original contributions presented in this study are included in the article/Supplementary Material. Further inquiries can be directed to the corresponding author.

## Footnotes

1 Here, the number indicates the number of limbs; the string of characters indicates the limb type; R, P, S, and X denote revolute, prismatic, spherical, and compliant X-shaped pairs, respectively; and the sequence of characters in the string indicates the sequence of joint types encountered in the limb when moving from the frame to the distal link.

## Supplementary Materials

The following supporting information can be downloaded at: https://www.mdpi.com/article/10.3390/biomimetics10120814/s1. This link includes the GeoGebra program and videos. (This consists of the GeoGebra program for building the digital twins shown in Figure 5, Figure 6, Figure 7 and Figure 8 and Figure 10, and four videos that show the neutral position (Figure 5) from different points of view, and animations of the axial rotation (Figure 8), the flexion/extension (Figure 6), and the lateral bending (Figure 7).)

## Abbreviations

The following abbreviations are used in this manuscript:

ALS	Amyotrophic lateral sclerosis
HNC	Head and neck cancer
ROM	Range of motion
CSI	Cervical spine injury
C1, …, C7	Vertebrae of the cervical spine (see Figure 1b)
O	Occipital bone
WAD	Wearable assistive device
SM	Serial mechanism
PM	Parallel mechanism
DOF	Degree of freedom
P	Prismatic pair or only the adjective “prismatic”
R	Revolute pair or only the adjective “revolute”
S	Spherical pair or only the adjective “spherical”
U	Universal joint
SMC	Spherical motion center
IPA	Inverse position analysis
FPA	Forward position analysis
IK	Instantaneous kinematics
IIK	Inverse instantaneous kinematics
FIK	Forward instantaneous kinematics
OW	Orientation workspace
IAR	Instantaneous axis of rotation
ISA	Instantaneous screw axis
